# The STING inhibitor (ISD-017) reduces glomerulonephritis in 129.B6.*Fcgr2b*-deficient mice

**DOI:** 10.1038/s41598-024-61597-z

**Published:** 2024-05-14

**Authors:** Isara Alee, Papasara Chantawichitwong, Asada Leelahavanichkul, Søren R. Paludan, Trairak Pisitkun, Prapaporn Pisitkun

**Affiliations:** 1 Center of Excellence in Systems Biology, Faculty of Medicine, Chulalongkorn University, Bangkok, Thailand; 2 Medical Sciences Program, Faculty of Medicine, Chulalongkorn University, Bangkok, Thailand; 3 Graduated Program in Molecular Medicine, Faculty of Science, Mahidol University, Salaya, Thailand; 4 Center of Excellence in Translational Research in Inflammation and Immunology (CETRII), Department of Microbiology, Faculty of Medicine, Chulalongkorn University, Bangkok, Thailand; 5 Department of Microbiology, Faculty of Medicine, Chulalongkorn University, Bangkok, Thailand; 6 Department of Biomedicine, Aarhus University, Aarhus, Denmark; 7 Epithelial Systems Biology Laboratory, National Heart, Lung, and Blood Institute, National Institutes of Health, Bethesda, MD USA; 8 Division of Allergy, Immunology, and Rheumatology, Department of Medicine, Faculty of Medicine Ramathibodi Hospital, Mahidol University, Bangkok, Thailand

**Keywords:** Lupus nephritis, Sting, SLE, Sting inhibitor, ISD017, Lupus nephritis, Autoimmunity

## Abstract

The absence of stimulator of interferon genes (STING) in 129.B6.*Fcgr2b*-deficient mice rescue lupus phenotypes. The administration of a STING inhibitor (ISD017) into the young 129.B6.*Fcgr2b*-deficient mice prevents lupus nephritis development. This study mainly aimed to evaluate the effects of STING inhibition (ISD107) on established SLE in mice to prove that ISD017 could be a good therapeutic drug to reverse the already set-up autoimmunity and kidney impairment. Twenty-four-week-old *Fcgr2b*-deficient mice were treated with cyclophosphamide (25 mg/kg, intraperitoneal, once per week), ISD017 (10 mg/kg, intraperitoneal, three times per week), or control vehicle for 8 weeks, and were analyzed for phenotypes. Both ISD017 and cyclophosphamide treatment increased long-term survival and reduced the severity of glomerulonephritis in *Fcgr2b*-deficient mice. While cyclophosphamide reduced activated B cells (B220^+^GL-7^+^), ISD017 decreased activated T cells (CD4^+^CD69^+^) and neutrophils (Ly6c^+^Ly6g^+^) in *Fcgr2b*-deficient mice. In addition, ISD017 reduced IL-1β and interferon-inducible genes. In summary, ISD017 treatment in symptomatic 129.B6.*Fcgr2b*-deficient mice reduced the severity of glomerulonephritis and increased long-term survival. ISD017 worked comparably to cyclophosphamide for treating lupus nephritis in 129.B6.*Fcgr2b*-deficient mice. ISD017 reduced activated T cells and neutrophils, while cyclophosphamide targeted activated B cells. These results suggested that STING inhibitors can potentially be a new therapeutic drug for treating lupus.

## Introduction

Systemic lupus erythematosus (SLE) is a chronic autoimmune disease mediated by genetic susceptibility triggered by persistent immune activation leading to the loss of self-tolerance and the production of antibodies against nuclear antigens^1^. The increased type I interferon (IFN-type I) activity or abnormal signaling has been identified as an essential pathogenic mechanism in SLE^2^. The binding of these autoantibodies to nuclear antigens forms immune complexes and subsequently induces type I IFN production. The capture of immune complexes by plasmacytoid dendritic cells enhances type I IFN production and the development of autoreactive B cells^3^. With the help of T follicular helper T cells, the autoreactive B cells increase autoantibody production^4^. The circulating immune complexes can accumulate throughout the organs, leading to complement activation, inflammation, and damage. The intricate link between the immune complexes and type I interferon signaling highlights the DNA sensing mechanisms, such as those mediated by the STING pathway, and the autoimmune responses observed in SLE.

The presence of autoantibodies in SLE indicates a defect in B cell tolerance. The autoreactive B cells could differentiate and receive help from T follicular cells in the germinal center (T cell-dependent) and become long-lived plasma cells. In addition, without T cell help, autoreactive B cells can differentiate into short-lived plasma cells outside the germinal center (T cell-independent)^5^.

The etiology of SLE involves both genetic and environmental factors. Genetic polymorphisms influence the clinical response to SLE treatment^6^. Patients with high interferon signatures respond well to treatment with anifrolumab (anti-interferon type 1 receptor)^7,8^. In addition, anti-BAFF antibodies show significant efficacy in treating SLE^8^. These data suggest that the heterogeneous nature of SLE requires various targeted treatments. The mechanisms of targeted therapy need to be explored to determine how the drug benefits SLE patients.

Deficiency of Fc gamma receptor IIb (*Fcgr2b*), the sole inhibitory receptor in the FcGR family, is one of the genetic factors implicated in lupus disease^9,10^. *Fcgr2b*-deficient mice have shown elevated type I IFN signaling, leading to increased autoantibody production and SLE-like symptoms in mice by 6–8 months in the 129/Sv B6 background but not in the BALB/C background^9,11^. The *Fcgr2b* gene is located on chromosome 1, which contains several lupus susceptibility genes in humans and mice^12,13^. The backcrossed of the original 129. *Fcgr2b*^*−/−*^ mice and C57BL/6 mice were created to study the effect of linked genes on autoimmune development^14^. The C57BL/6. *Fcgr2b*^*−/−*^_129_. *Slamf*_B6_ congenic mice and C57BL/6. *Slamf*_129_ mice developed mild disease symptoms compared to the original 129 mice. *Fcgr2b*^*−/−*^ mice^15^. The interaction of Fcgr2b and 129 strain-derived SLAM family proteins enhanced autoimmunity and spontaneous germinal centers^14,15^**,** while *Fcgr2b*-deficient mice generated using B6 ES cells did not develop overt lupus phenotypes^16^. The interaction between multiple genetic loci in 129 backgrounds suggested the similar complexity of lupus susceptibility to human SLE.

Multiple genes in the linked locus of *Fcgr2b* contribute to SLE susceptibility. The Nba2 intervals, including Fcgr, SLAM, and interferon-inducible genes, enhance autoantibody production and renal disease^17^. The IFN-dependent phenotype of the *Fcgr2b*-deficient mice in this study could be related to the linkage region. These findings suggested the importance of multiple genetic loci in chromosome 1 of the 129/Sv background, with the *Fcgr2b* region involved in lupus development^12^.

Several roles of *FCGR2B* in human SLE have been studied. The meta-analysis of *FCGR2B* polymorphisms shows the association between *FCGR2B* (rs1050501) and SLE susceptibility under the recessive genotypic model of the C allele in the overall population^18^, and low copy numbers of *FCGR2B are* associated with SLE susceptibility^19^. The polymorphisms of *FCGR2B*-T232 in the transmembrane domain reduce the lateral mobility and inhibitory function of FCGR2B^20^. In addition, polymorphisms in the *FCGR2B-FCRLA* locus are associated with non-responders to intravenous cyclophosphamide treatment for lupus nephritis^21^. Thus, the *Fcgr2b*-deficient lupus mouse is a relevant model for studying human SLE pathogenesis.

Stimulator of interferon genes (STING) is induced by cGAS when it recognizes dsDNA, increasing type I IFN production and showing an emerging role in lupus pathogenesis^22^. A gain-of-function STING mutation in humans causes SAVI (STING-associated vasculopathy with onset in infancy), a disease of interferonopathy^23^. *Sting* deficiency partially rescues perinatal lethality and reduces inflammation in Rnaseh2 knock-in mice, showing increased interferon-stimulated gene expression^24^. In addition, *Trex1*-deficient mice with autoimmune phenotypes and myocarditis survived without Sting^25,26^. These data suggested that STING signaling contributed to non-lupus autoimmune mouse models.

TLR-mediated mechanisms play roles in the *MRL. Fas*^*lpr*^ lupus-prone mice^27^, While the *MRL. Fas*^*lpr*^ mice develop fatal glomerulonephritis, plasmacytoid DC activation, activated T- and B- cells, and anti-dsDNA production without *Tlr9*^28^; the absence of *Tlr7* reduces the production of autoantibodies to RNA antigens, T- and B-cell activation, and pDC activation^28^. These data suggested differential roles of Toll-like receptors in lupus-liked phenotypes of the *MRL. Fas*^*lpr*^ mice. However, *Sting* deficiency in *MRL. Fas*^*lpr*^ lupus-prone mice have accelerated the severity of lupus^29^.

Unlike the *MRL. Fas*^*lpr*^ lupus-prone mice, the *Fcgr2b*-deficient mice show a reduction of anti-dsDNA autoantibodies when *Tlr9* is absent^30^. At the same time, the overexpression of *Tlr7* contributes to lupus phenotype in *Yaa* (Y-linked autoimmune acceleration) carrying mice^31,32^, *Tlr7* deficiency in *Fcgr2b* -deficient *Yaa* mice reduces plasma cell expansion and T cell activation^33^. Furthermore, STING-mediated signaling initiates lupus development in *Fcgr2b*-deficient lupus mice by expanding dendritic cells^11^. The role of STING in the lupus mouse model may differ in the context of pathway-mediated type I IFN signaling.

Therefore, studies utilizing animal models of lupus are essential for further understanding the pathogenesis of SLE, leading to the development of effective treatments for patients. Previous studies have utilized a newly developed STING inhibitor molecule (ISD017) in lupus mouse models lacking the *Fcgr2b* gene. Administering ISD017 before the onset of anti-nuclear antibodies and lupus symptoms effectively reduced glomerulonephritis and autoantibody production in *Fcgr2b*-deficient mice^34^. These findings support the potential application of ISD017 in the treatment of SLE. Administering the drug to high-risk individuals before SLE symptoms may not be practical in a clinical setting. However, to apply ISD017 effectively in treating SLE, studies investigating its efficacy in lupus mouse models that already exhibit symptoms or signs of the disease will provide proof of concept for using ISD017 in real-life clinical settings.

Therefore, this study mainly aimed to evaluate the effects of STING inhibition (ISD107) on established SLE in mice that showed autoantibodies and proteinuria to prove that ISD017 could be a therapeutic drug for the already set-up autoimmunity and kidney impairment. The researcher will use lupus laboratory animal models, specifically 6- to 8-month-old *Fcgr2b*-deficient lupus mice presenting high levels of autoantibodies and proteinuria. The efficacy and underlying mechanisms of ISD017 will be compared to the standard immunosuppressive treatment, cyclophosphamide, commonly used for treating lupus nephritis (LN) in SLE.

## Methods

### Animal model and experiment

*Fcgr2b-deficient* mice on the 129/C57BL/6 background (MGI Cat# 2448997, RRID: MGI:2448997) were obtained from Dr. Bolland (NIH, Maryland, USA). The *Fcgr2b* deletion was constructed on a 129 Sv/B6 hybrid background and backcrossed to C57BL/6 for 12 generations^35^. The 129.B6. *Fcgr2b*-deficient mice were bred together to maintain the original 129. *Fcgr2b*-deficient mice. The littermates of 129.B6. *Fcgr2b*-deficient mice, both male and female mice, were followed up to the age of 6 months and screened for anti-dsDNA to confirm the development of the lupus phenotype before randomly enrolling into three groups. The three treatments were cyclophosphamide (CYC) (25 mg/kg, intraperitoneal, once per week)^36^, ISD-017 (10 mg/kg, intraperitoneal, three times per week)^34^, or control vehicle (PBS), and the treatment period lasted for 8 weeks. We assessed the anti-double-stranded DNA (anti-dsDNA) in all groups of mice before initiating treatment and after treatment for 8 weeks. The survival of the mice was observed during the treatment period. The number of laboratory mouse cohorts at the initial treatment of the ISD-017, CYC, and PBS groups was 15, 9, and 20 mice, respectively. Mice were monitored throughout treatment, and those that expired prematurely were excluded from analysis. After 8 weeks of treatment, the surviving mice were humanely euthanized, and blood, kidney, spleen, and urine were collected for further analysis. Mice were bred and housed at the Faculty of Medicine, Chulalongkorn University. All experiments were performed with the approval of the Animal Experimentation Ethics Committee of Chulalongkorn University Medical School with all relevant institutional guidelines (research no. 003/2563). All methods were performed following the ARRIVE guidelines.

### ISD017 (STING antagonist)

ISD 017 was derived from the conserved hemagglutinin fusion peptide (FP) that antagonizes type I interferon production induced by membrane fusion or influenza A virus^37^. ISD017 is specific to cGAS and impacts dsDNA sensing without impacting other nucleic acids^34^. In addition, ISD017 inhibited dsDNA activation of both type I IFN and TNF production^34^. To dissolve ISD017, a panel of solvents was tested, namely, H_2_O, 0.9% NaCl, TBS (pH 7.3), PBS (pH 7.4), HEPES, and PBS (pH 7.4) + 1 M NaOH. For the latter, which was most successful, 194 µL of PBS mixed with 6 µL of 1 M NaOH was added to 1 mg of ISD017.

### Detection of anti-nuclear antibodies (ANA)

The sera (1:400) of *Fcgr2b*-deficient mice were diluted at 8 months, and wild-type mice were tested for ANA as described^11^. In short, diluted serum (30 µL) was added to the Hep-2 cell-coated slide, and phosphate-buffered saline (PBS) was used as a negative control, followed by incubation for 30 min. Then, the cells were washed with PBS 2 times and incubated with 30 µL of goat anti-mouse IgG–Alexa (1:500) (Abcam, Cambridge, MA, USA; cat. FA 1512-1010-1) for 30 min and washed with PBS. Finally, the slides were fixed and analyzed under a fluorescence microscope. The researcher will be blinded and grade the intensity as 4 = maximal fluorescence (brilliant yellow-green), 3 = less brilliant (yellow-green fluorescence), 2 = definite (dull yellow-green), and 1 = very dim (subdued fluorescence).

### Detection of anti-dsDNA antibodies

The quantitative ELISA for anti-dsDNA was performed from the sera collected two months after treatment using the previous protocol^11^. In short, ds-DNA in carbonate coating buffer (10 µL/mL) was coated on a 96-well plate at 4 °C overnight. Then, the plate was washed with 0.05% Tween-20/PBS (washing buffer) 5 times, and 100 µL of blocking solution (10% BSA/Tween-20/PBS) was added and incubated at room temperature for 90 min. Afterward, the cells were washed with washing buffer 5 times, 100 µL of serum (1:100) was added, and the cells were incubated at 37 °C for 60 min and washed 5 times. The anti-dsDNA antibody was detected using goat anti-mouse IgG conjugated with HRP and ABTS peroxidase substrate; then, the absorption value was measured at a wavelength of 450 nm.

### Flow cytometry analysis

The spleens were dissected and passed through a 70-µm filter. Splenocytes were placed in PBS solution and centrifuged at 1500 rpm for 5 min to precipitate the cells. The pellet was treated with ACK buffer (NH4Cl, KHCO3, and EDTA) to eliminate erythrocytes. The staining protocol was previously described^11^ using the following antibodies: anti-CD4 (clone: GK1. 5; cat. 100423), CD8 (clone: 53–6. 7; cat. 100708), CD62L (clone: MEL-14; cat. 104417), CD44 (clone: IM7; cat. 103035), CD3ε (clone: 145-2C11; cat. 100312), ICOS (clone: C398.4A; cat. 313517), CD11c (clone: N418; cat. 117312), B220 (clone: RA3-6B2; cat. 103222), CD11b (clone: M1/70; cat. 101228), Ly6c (clone: HK1.4; cat. 128022), Ly6g (clone 1A8; cat. 127608), I-Ab (clone: AF6-120.1; cat. 116406), PDCA-1 (clone: 129c1; cat. 127103), CD80 (clone: 16-10A1; cat. 104733), GL7 (clone: GL7; cat. 144604), and CD138 (clone: 281–2; cat. 142506) (Biolegend, San Diego, CA, USA). The stained cells were dissolved in 1% paraformaldehyde/PBS for analysis.

The analysis of serum cytokines was performed using the LEGENDplex™ Mouse Inflammation Panel kit (Biolegend, San Diego, CA, USA; Cat. No. 740446) following the manufacturer's instructions. Flow cytometry was performed using an LSR II flow cytometer (BD Biosciences, USA) and analyzed using FlowJo software (USA). LEGENDplexTM Data Analysis Software was used to analyze the cytokine data.

### Measurement of creatinine

The evaluation of creatinine in blood and urine was performed using a QuantiChrom™ Creatinine Assay Kit (DICT-500) following the manufacturer's description. The serum was extracted from the blood, and the urine was diluted (1:50). Next, 30 µL of the serum and the diluted urine in PBS were dripped into a 96-well plate. Then, 200 µL of the working reagent, a 1:1 ratio of reagents A and B, was added to each well and gently mixed. The light absorption of each sample was measured at 1 min and 5 min using a spectrophotometer at a wavelength range of 490–530 nm (with peak absorbance at 510 nm). The result is calculated using a specific formula (as below: n = dilution factor) to evaluate the creatinine levels in the blood and urine samples.

$${\text{Creatinine}}\;{\text{concentration}} = \left( {{\text{OD}}\;{\text{sample}}_{5} - {\text{OD}}\;{\text{sample}}_{1} } \right) \times {\text{n}} \times 2\;\left( {{\text{mg}}/{\text{dL}}} \right)/\left( {{\text{OD}}\;{\text{standard}}_{5} - {\text{OD}}\;{\text{standard}}_{1} } \right)$$

### Histopathology

Kidney tissues were fixed in 10% neutral buffered formalin for 24 h. Five-micrometer-thick paraffin-embedded (FFPE) sections were stained with hematoxylin and eosin. The pathology grading from kidney sections was blinded by an experienced researcher. As previously described, the histology scores were reported as glomerular and interstitial scores^38–39^. In brief, glomerular scores were defined as 0 = normal; 1 = focal, mild, or early proliferative; 2 = moderate or definite proliferative; 3 = diffuse and focal or diffuse proliferative; 4 = severe diffuse proliferative with crescent/sclerosis, and interstitial scores were defined as 0 = normal; 1 = focal or small pockets (10–15 cells) of mononuclear cells (MNC); 2 = focal infiltrates (15–30 cells); 3 = multifocal extensive infiltrates with necrosis; 4 = multifocal or diffuse and extensive with necrosis.

### Immunofluorescence

The frozen renal sections were first fixed in acetone and then blocked with 1% BSA in PBS. Next, the sections were stained using FITC-conjugated goat anti-mouse IgG (cat. 4408) or anti-C3c FITC (cat. Ab4212) (Abcam, Cambridge, MA, USA). Afterward, the samples were stained with DAPI (4ʹ,6-Diamidino-2-Phenylindole, Dihydrochloride) (Thermo Fisher Scientific, MA, USA) for 5 min in the dark at room temperature. Following staining, the slides underwent three washes and were subsequently mounted with ProLongTM Diamond Antifade Mountant (Invitrogen, CA, USA). The fluorescent signals were then visualized using ZEISS LSM 800 with Airyscan (Carl Zeiss, Germany). The fluorescence intensity was quantified using ZEISS ZEN Microscope Software (Carl Zeiss, Germany).

### Measurement of Total IgG

The 96-well plates were coated with 100 µL of Goat Anti-Rabbit IgG (H + L) (AffiniPure™) and incubated at 4 °C overnight. The supernatant was removed and washed with 1X PBST three times. Subsequently, the samples were diluted 1:30,000 in 1X PBST. A volume of 100 µL of each diluted sample was then added to the wells and incubated at 37 °C for 1 h. Afterward, the clear supernatant was removed, and the wells were washed three times with 1X PBST. Subsequently, 100 µL of the secondary antibody (Peroxidase AffiniPure™ Goat Anti-Rabbit IgG (H + L)), diluted 1:8000 in 1X PBST, was added to each well. The samples were then incubated at 37 °C for 1 h. After the supernatant was removed, 100 µL of the substrate was added to each well and then incubated at room temperature for 20 min in the dark. Finally, add 2N H_2_SO_4_ to stop the reaction and measure the absorbance value at 492 nm using a Cytation™ 5 machine.

### Statistical analysis

For multiple comparisons, two-tailed ANOVA was used, and p-values were calculated to determine the statistical significance of differences among groups using GraphPad Prism software (version 7, San Diego, CA, USA). Data will be expressed for all experiments as the mean ± s.e.m., and p values < 0.05 were considered statistically significant: *p ≤ 0.05 and **p ≤ 0.01.

## Results

### ISD017 significantly increased the survival of symptomatic *Fcgr2b*^−/−^ mice

**Figure 1:**
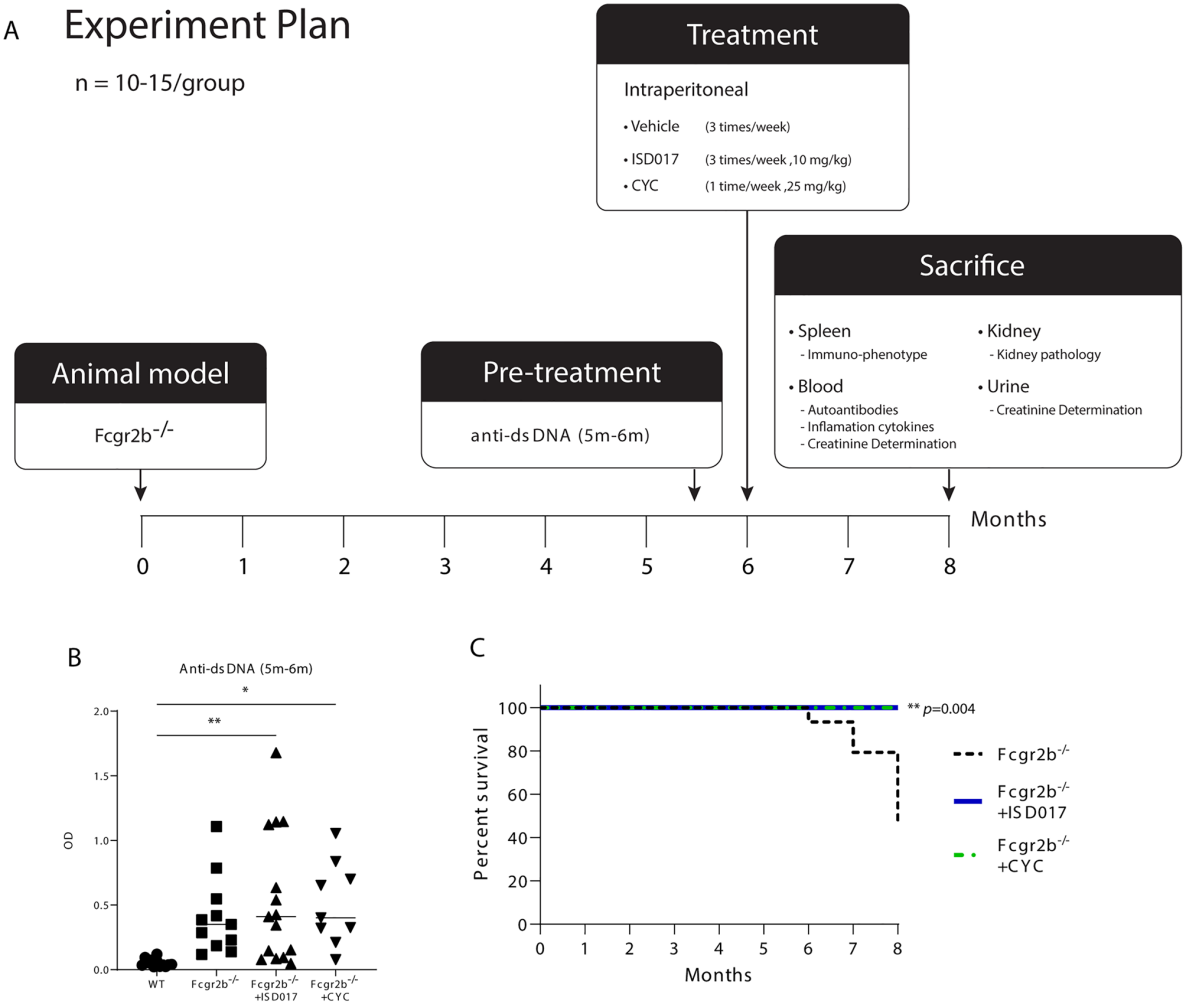
ISD-017 significantly increased the survival of symptomatic *Fcgr2b*^*−/−*^ mice. (**A**) Chart of the experimental treatment in *Fcgr2b*^*−/−*^ mice showing the timeline of intraperitoneal injection of ISD017 (25 mg/mL, 3 times/week) (N = 15), cyclophosphamide (CYC) (10 mg/mL, 1 time/week) (N = 9), or PBS control into 6-month-old mice (N = 20) for 2 months, and then the immunophenotypes were observed. (**B**) Baseline anti-dsDNA of the 6-month-old *Fcgr2b*^*−/−*^ mice detected by ELISA (N = 9–15 per group). (**C**) The survival curve of the mice was observed for up to 8 months (N = 9–15 per group). The comparison between the *Fcgr2b*^*−/−*^ vs *Fcgr2b*^*−/−*^ + ISD017 and the *Fcgr2b*^*−/−*^ vs *Fcgr2b*^*−/−*^ + CYC showed p-value = 0.004. Error bars indicate SEM; *p < 0.05, **p < 0.01, and ***p < 0.001.

We intraperitoneally injected symptomatic *Fcgr2b*^*−/−*^ mice (5–6 months old) with ISD017 or cyclophosphamide to determine whether ISD017 can be used as a treatment for overt lupus phenotypes compared to the standard treatment, cyclophosphamide (Fig. 1A). We screened the level of anti-dsDNA in the experimental mice and randomly assigned the treatment groups. The level of anti-dsDNA between the *Fcgr2b*^*−/−*^ and treatment groups did not differ (Fig. 1B). The survival of the mice was observed during the treatment period. Upon evaluating the viability of laboratory mouse cohorts, all of the mice in the ISD017 treatment (N = 15) and CYC treatment (N = 9) groups survived. However, only 11 out of 20 mice in the PBS treatment group survived. We observed that *Fcgr2b*^*−/−*^ mice survived significantly longer in the ISD017- and CYC-treated mice than the non-treated *Fcgr2b*^*−/−*^ mice (p = 0.004). All ISD017- and CYC-treated *Fcgr2b*^*−/−*^ mice survived throughout the treatment, while 55% of the non-treated *Fcgr2b*^*−/−*^ mice died (Fig. 1C).

### ISD017 reduced the severity of glomerulonephritis in *Fcgr2b*^−/−^ mice

**Figure 2:**
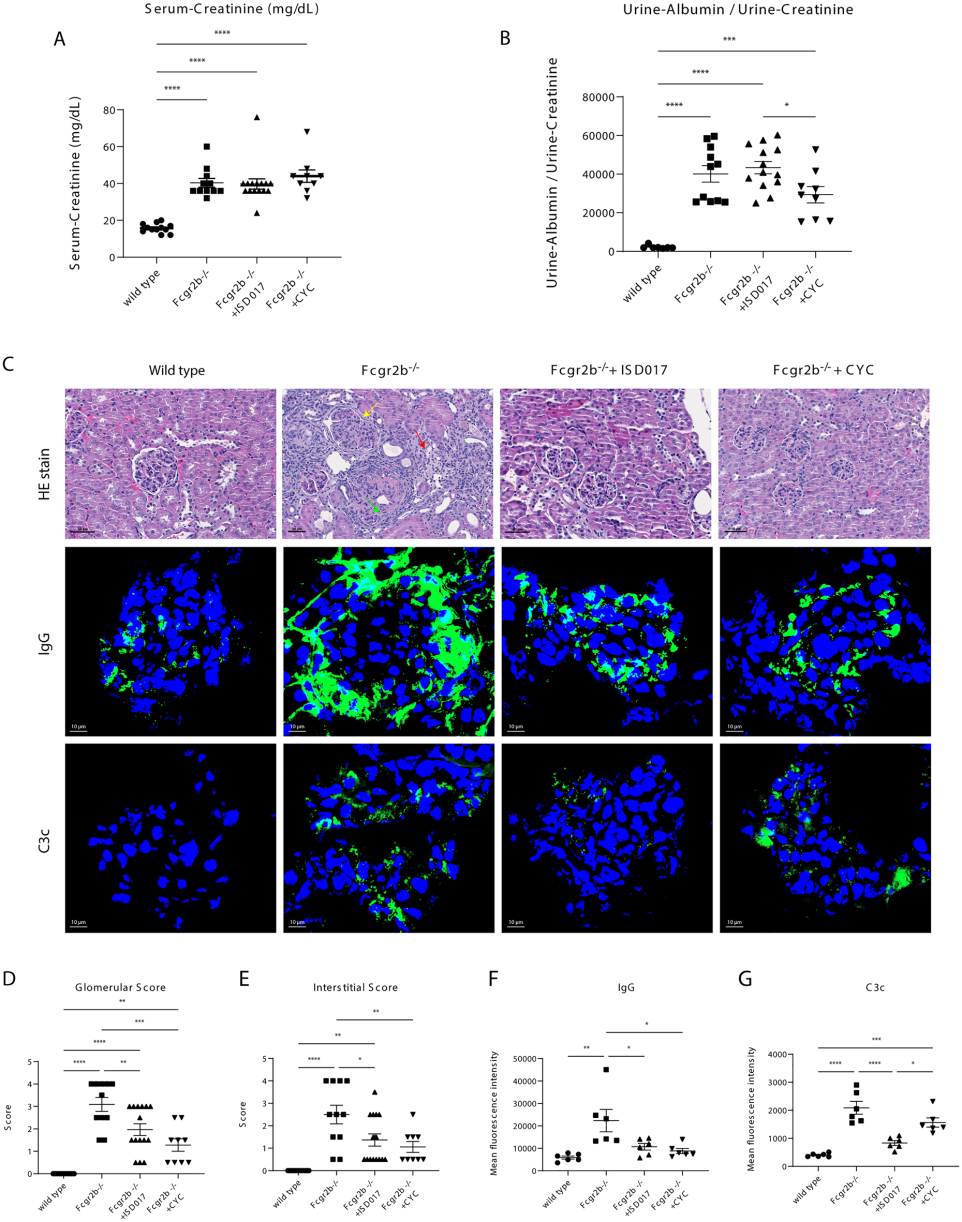
ISD017 reduced the severity of glomerulonephritis in *Fcgr2b*^*−/−*^ mice. (**A**) Serum creatinine of WT, *Fcgr2b*^*−/−*^ + PBS control, *Fcgr2b*^*−/−*^ + ISD017, and *Fcgr2b*^*−/−*^ + CYC mice and (**B**) urine albumin/urine creatinine of WT, *Fcgr2b*^*−/−*^ + *PBS control, Fcgr2b*^*−/−*^ + *ISD017, and Fcgr2b*^*−/−*^ + *CYC mice* were detected after treatment with ISD-017, CYC, or PBS for 2 months. (N = 9–15 per group). (**C**) Kidney sections from WT, control *Fcgr2b*^*−/−*^ mice (8 months old), and treated *Fcgr2b*^*−/−*^ mice with ISD017 or CYC were stained with H&E (top row). The kidney histopathology of *Fcgr2b*^*−/−*^ control showed fibrocellular crescent (green arrow), glomerulosclerosis (yellow arrow), and interstitial infiltration (red arrow). The data are representative of 9–15 mice per group (scale bar = 50 µm). The immunofluorescence (IF) staining of IgG (middle row) and C3c (lower row). The data are representative of 6 mice per group (scale bar = 10 µm). (**D**) Glomerular and (**E**) interstitial scores of kidney sections were blindly graded (N = 9–15 per group). (**F**,**G**) The mean fluorescence intensity of IgG (F) and C3c (**G**) were analyzed (N = 6 per group). Data are shown as the mean ± SEM; *p < 0.05, **p < 0.01 and ***p < 0.001.

**Figure 3:**
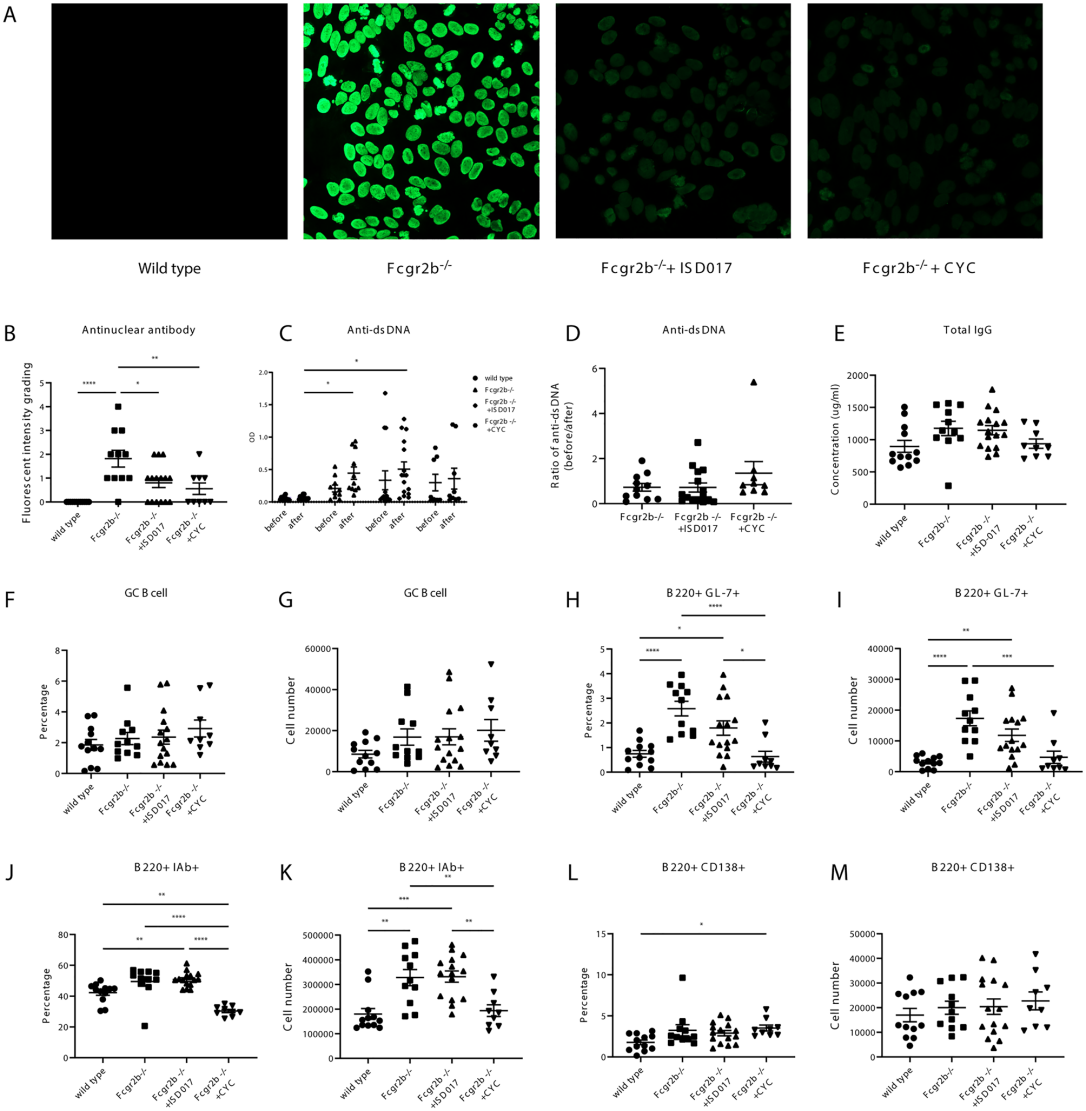
CYC decreased spontaneously activated B cells. Serum from the treated and control *Fcgr2b*^*−/−*^ mice showed (**A**) the anti-nuclear antibody (ANA) by immunofluorescence staining of Hep-2 cells and (**B**) a semiquantitative level of the anti-nuclear antibody (ANA) graded by fluorescence intensity, (**C**) anti-dsDNA (Before and after treatment), and (**D**) The OD ratio of anti-dsDNA (before/after treatment) from the serum of the treated *Fcgr2b*^*−/−*^ mice and control *Fcgr2b*^*−/−*^ mice. (**E**) total IgG detected by ELISA (N = 9–15 per group). (**F**–**M**) Isolated splenocytes from *Fcgr2b*^*−/−*^ mice (8 months old) from ISD017- or CYC-treated and control *Fcgr2b*^*−/−*^ mice were analyzed by flow cytometry. (**F**) The percentage of GC B cells and (**G**) cell numbers of GC B cells are shown. (**H**) The percentage of B220^+^GL-7^+^and (**I**) cell numbers of B220^+^GL-7^+^ are shown. (**J**) The percentage of B220^+^IAb^+^ and (**K**) cell numbers of B220^+^IAb^+^ are shown. (**L**) The percentage of B220^+^CD138^+^ (plasma cells) and (**M**) cell numbers of B220^+^CD138^+^ are shown (N = 9–15 per group). Data are shown as the mean ± SEM; *p < 0.05, **p < 0.01 and ***p < 0.001.

The *Fcgr2b*^*−/−*^ mice showed serum creatinine levels higher than WT mice at the same age (Fig. 2A). The mice treated with ISD017 and CYC showed comparable serum creatinine levels with non-treated mice (Fig. 2A); however, only CYC reduced the urine protein/creatinine ratio (Fig. 2B). Next, the histological findings of the kidneys by H&E staining showed that *Fcgr2b*^*−/−*^ mice developed fibrocellular crescent, glomerulosclerosis, and interstitial infiltration, while the treated *Fcgr2b*^*−/−*^ mice with ISD017 and CYC had less severe disease (Fig. 2C, top row). The kidney glomerular scores (Fig. 2D) and interstitial scores (Fig. 2E) in both treatment groups were significantly decreased compared to those in non-treated *Fcgr2b*^*−/−*^ mice. The immunofluorescence staining of IgG (Fig. 3C, middle row, and 3F) in the ISD017- and CYC-treated *Fcgr2b*^*−/−*^ mice were reduced. However, the C3c staining in the kidney was significantly reduced in the ISD017-treated mice (Fig. 3C, bottom row, and 3G).

### CYC decreased spontaneously activated B cells

The immunofluorescence staining using Hep-2 cells identified the anti-nuclear antibody (ANA) (Fig. 3A). The ANA patterns of serum from the *Fcgr2b*^*−/−*^ mice showed homogenous and fine-speckled patterns, which did not change by the treatment (Fig. 3A). However, the intensity of ANA staining was reduced in ISD017 and CYC-treated *Fcgr2b*^*−/−*^ mice. The analysis of ANA levels revealed that the untreated *Fcgr2b*^*−/−*^ group had significantly higher values than WT mice and the two treatment groups (Fig. 3B). However, no statistically significant difference was observed in the anti-dsDNA level after the treatment among the *Fcgr2b*^*−/−*^ groups (Fig. 3C). After the normalization of anti-dsDNA, the ratio of anti-dsDNA (before/after) did not show a difference between treated groups and non-treated *Fcgr2b*^*−/−*^ mice (Fig. 3D). Next, we tested the total IgG to see the treatment effect. The CYC-treated *Fcgr2b*^*−/−*^ mice tend to have lower IgG levels but did not reach the statistical significance (Fig. 3E). The expansion of spontaneous germinal center B cells has been shown in *Fcgr2b*^*−/−*^ mice^40^, and germinal center formation is required to exclude autoreactive B cells^41^. Then, we identified germinal center B cells using GL-7^hi^FAS^hi^ staining (Supplementary Fig. 1A) and did not detect the reduction of this subset by the ISD017 and CYC treatment (Fig. 3F,G). Next, we looked at the activating state of B cells by using the GL-7^+^ and IAb^+^. However, GL-7^+^ B cells significantly decreased in percentages (Fig. 3H) and absolute cell numbers (Fig. 3I) in CYC-treated *Fcgr2b*^*−/−*^ mice. Similarly, the percentage (Fig. 3J) and total cell numbers (Fig. 3K) of B220^+^IAb^+^ cells were significantly lower in the CYC-treated mice. However, no significant difference was found between the non-treated and ISD-017-treated *Fcgr2b*^*−/−*^ mice. The B220^+^ cells showed a comparable mean fluorescence intensity (MFI) of IAb among all groups (Supplementary Fig. 1B). Notably, the expression of plasma cells showed no difference among the three *Fcgr2b*^*−/−*^ groups in percentage (Fig. 3L) and absolute cell numbers (Fig. 3M).

### ISD017 restricted the expansion of activated T cells

**Figure 4:**
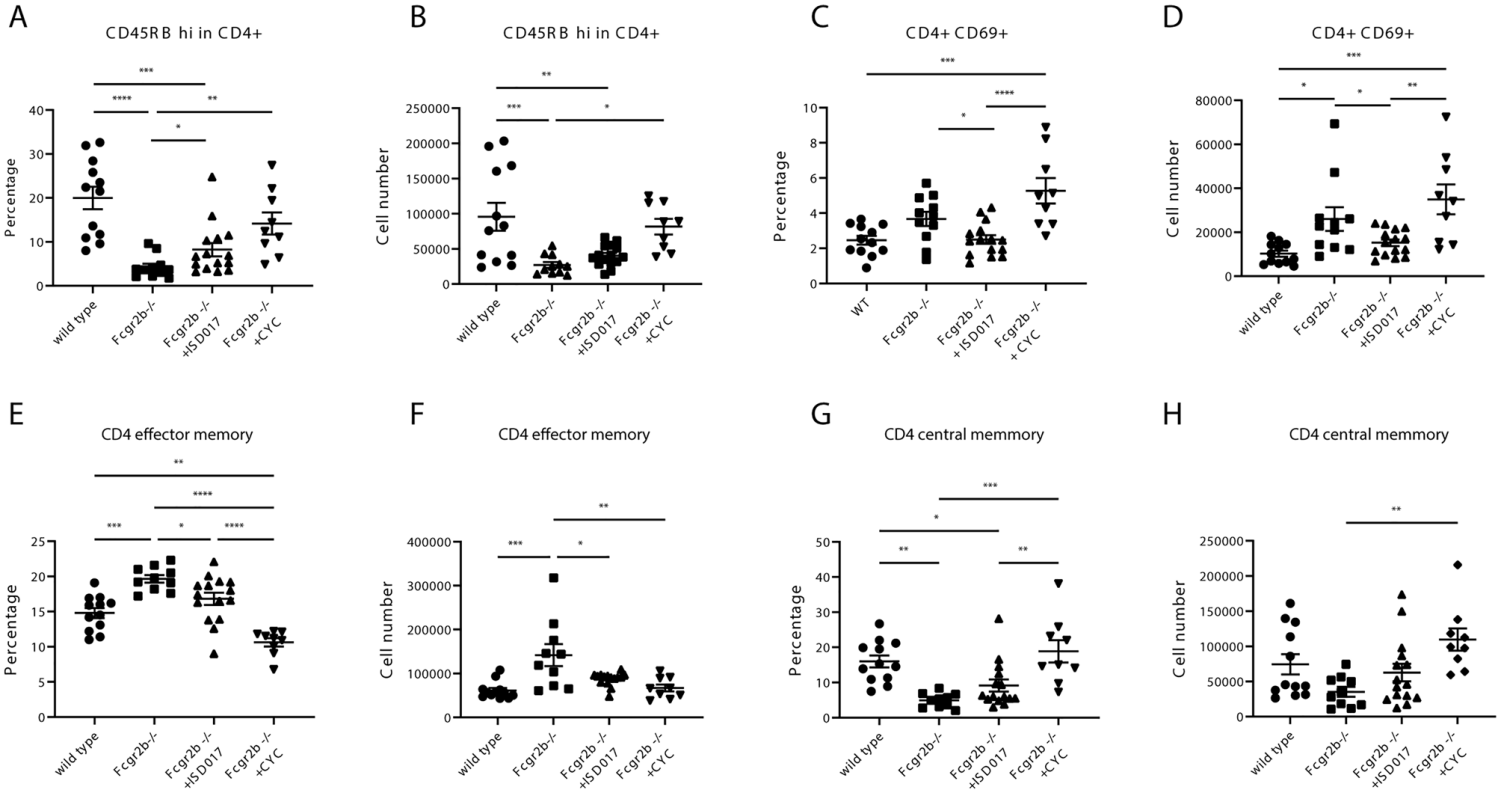
ISD017 restricted the expansion of activated T cells. (**A**–**H**) Isolated splenocytes from *Fcgr2b*^*−/−*^ mice (8 months old) from ISD017- or CYC-treated and PBS-treated control *Fcgr2b*^*−/−*^ mice were analyzed by flow cytometry. (**A**) The percentage of CD4^+^CD45RB^hi^ (naïve) and (**B**) cell numbers of CD4^+^CD45RB^hi^ are shown. (**C**) The percentage of CD4^+^CD69^+^ (activated T cells) and (**D**) cell numbers of CD4^+^CD69^+^ are shown. (**E**) The percentage of CD4^+^CD44^+^CD62L^-^ (T effector memory or T_EM_) and (**F**) cell numbers of CD4^+^CD44^+^CD62L^-^ (T effector memory or T_EM_) are shown. (**G**) The percentage of CD4^+^CD44^+^CD62L^+^ (T central memory or T_CM_) and (**H**) cell numbers of CD4^+^CD44^+^CD62L^+^ (T central memory or T_CM_) are shown (N = 9–15 per group). Data are shown as the mean ± SEM; *p < 0.05, **p < 0.01 and ***p < 0.001.

We investigated the targeted effector cells of ISD017 treatment compared to CYC treatment. CD4^+^CD45RB^hi^ cells are described as naïve effector T cells^42^. The ISD017- and CYC-treated *Fcgr2b*^*−/−*^ mice showed an increase in the percentage of CD4^+^CD45RB^hi^ cells compared to non-treated mice (Fig. 4A). However, only CYC treatment increased the absolute number of these cells (Fig. 4B). In addition, only ISD017 limited the expansion of activated T cells (CD4^+^CD69^+^) in *Fcgr2b*^*−/−*^ mice (Fig. 4C,D). Although ISD017 did not decrease the MFI of CD69 on CD4^+^ T cells compared to *Fcgr2b*^*−/−*^ control mice (Supplementary Fig. 1C), the CYC-treated mice showed significantly higher CD69 expression on CD4^+^ T cells than *Fcgr2b*^*−/−*^ control mice and ISD017 treated mice (Supplementary Fig. 1C). Next, we gated CD3^+^CD4^+^ on the CD44 and CD62L (Supplementary Fig. 1D) to identify the effector memory T cells (T_EM_ or CD44^+^CD62L^-^) and central memory T cells (T_CM_ or CD44^+^CD62L^+^). While both ISD017 and CYC treatment significantly reduced the number of CD4^+^CD44^+^CD62L^-^ (T_EM_) cells compared to that in non-treated *Fcgr2b*^*−/−*^ mice (Fig. 4E-4F), only CYC treatment increased the number of CD4^+^CD44^+^CD62L^+^ (T_CM_) cells (Fig. 4G-4H).

### ISD017 diminished the expansion of neutrophils and the production of IL-1β

**Figure 5:**
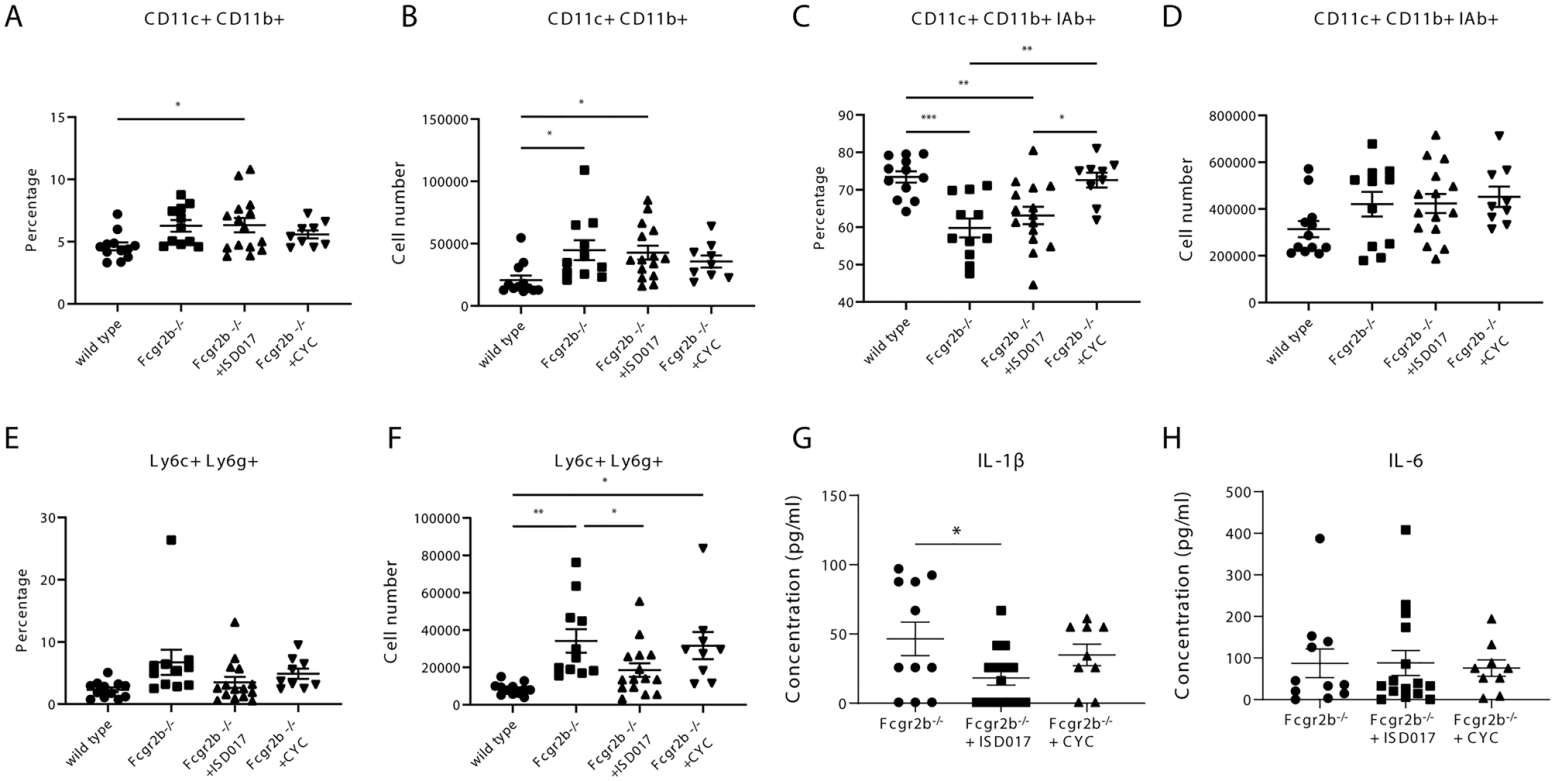
ISD017 diminished the expansion of neutrophils and the production of IL-1β. (**A**–**D**) Isolated splenocytes from *Fcgr2b*^*−/−*^ mice (8 months old) from ISD017- or CYC-treated and PBS-treated control *Fcgr2b*^*−/−*^ mice were analyzed by flow cytometry. (**A**) The percentage of CD11b^+^CD11c^+^and (**B**) cell numbers of CD11b^+^CD11c^+^ are shown. (**C**) The percentage of CD11b^+^CD11c^+^ IAb^+^ and (**D**) cell numbers of CD11b^+^CD11c^+^ are shown. (**E**) The percentage of Ly6c^+^Ly6g^+^ and (**F**) cell numbers of Ly6c^+^Ly6g^+^ are shown (N = 9–15 per group). (**G**,**H**) Serum from the treated and PBS-treated control *Fcgr2b*^*−/−*^ mice (8 months old) was tested for (**G**) IL-1β and (**H**) IL-6 (N = 9–15 per group). Data are shown as the mean ± SEM; *p < 0.05, **p < 0.01 and ***p < 0.001.

Innate immunity is critical for lupus development^43^. The expansion of myeloid dendritic cells and neutrophils in *Fcgr2b*^*−/−*^ mice have been previously described^11,40^. Then, we analyzed the treatment effect on myeloid dendritic cells (CD11b^+^CD11c^+^) (Fig. 5A,B) and did not detect the difference in the absolute number of this population (Fig. 5B). To better identify more specific mature dendritic cells, we analyzed the CD11b^+^CD11c^+^IAb^+^ cells (Fig. 5C,D) and did not identify the difference in the number of CD11b^+^CD11c^+^IAb^+^ cells among the *Fcgr2b*^*−/−*^ mice (Fig. 5D). Next, we identified the effect of treatment on neutrophils (Ly6c^+^Ly6g^+^) (Fig. 5E,F). Although ISD017 and CYC did not change the percentage of neutrophils (Fig. 5E), ISD017 significantly reduced the total number of neutrophils (Fig. 5F). Furthermore, ISD017 reduced serum IL-1β in the treated *Fcgr2b*^*−/−*^ mice (Fig. 5G) but did not change the IL-6 level (Fig. 5H).

### ISD017 reduced the expression of interferon-inducible genes in *Fcgr2b*^−/−^ mice

**Figure 6:**
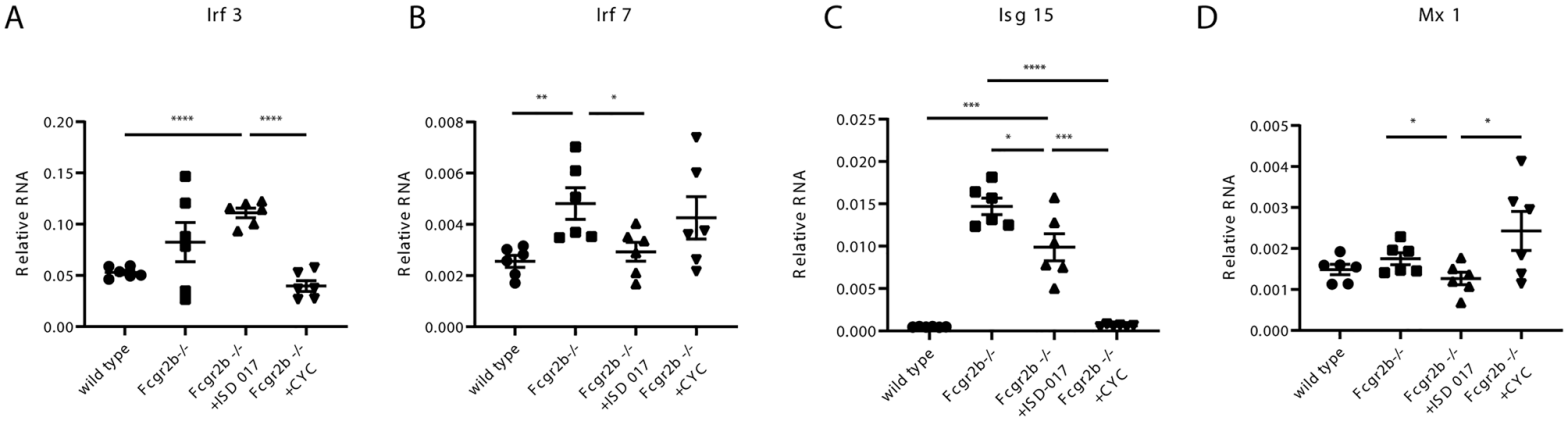
ISD017 reduced the expression of interferon-inducible genes in *Fcgr2b*^*−/−*^ mice. (**A**–**D**) The relative RNA expression (normalized to actin) of (**A**) *Irf3*, (**B**) *Irf7*, (**C**) *Isg*15, and (**D**) *Mx1* in the kidneys of WT, PBS-treated, ISD017-treated, and CYC-treated *Fcgr2b*^*−/−*^ mice at the age of 8 months is shown (N = 6 per group). Data are shown as the mean ± SEM; *p < 0.05, **p < 0.01 and ***p < 0.001. Due to the small sample size, analysis was performed using a Student's t-test to compare each pair.

The kidneys of *Fcgr2b*^*−/−*^ mice showed reduced expression of interferon-inducible genes (*Mx1* and *Isg15*) and interferon regulatory factors (*Irf3* and *Irf7*) in the absence of Sting^11^. Next, we tested whether ISD017 can effectively inhibit type I IFN signaling. We analyzed the expression of these genes in the kidneys of ISD017-treated *Fcgr2b*^*−/−*^ mice. ISD017 decreased the expression of *Irf7, Isg15* and *Mx1* (Fig. 6B–D) but not *Irf3* (Fig. 6A). However, CYC treatment suppressed the expression of *Irf3* (Fig. 6A) and *Isg15* (Fig. 6C) but not *Irf7* (Fig. 6B) and *Mx1* (Fig. 6D).

## Discussion

The increased levels of type I IFN and the upregulation of IFN-stimulated genes (ISGs) have been consistently observed in the blood and tissues of SLE patients and suggested severe disease activity^44^. This dysregulated production of type I IFN is thought to arise from the activation of plasmacytoid dendritic cells (pDCs) and the aberrant recognition of self-nucleic acids, leading to the induction of IFN-responsive genes and the subsequent promotion of autoimmune responses^45^. STING is an intracellular sensor that recognizes cytosolic DNA and triggers the production of type I IFN and proinflammatory cytokines^46^. Mice with enhanced type I IFN signaling or overexpression of STING exhibit lupus-like features. Conversely, suppressing STING signaling ameliorates lupus-like manifestations in several mouse models^11,47,48^. Inhibition of type I IFN using monoclonal antibodies or small molecules has shown promising results in reducing disease activity in SLE patients^7^. Similarly, STING inhibitors are being developed as potential therapeutic agents to modulate aberrant immune responses in SLE.

ISD017, a STING inhibitor, blocks the trafficking of STING from the ER to the Golgi, reducing inflammatory cytokines and type I interferon production^34^. ISD017 is specific to dsDNA-activated Sting but not MAVS or TRIF^34^. A previous study showed that ISD017 administration at a young age in *Fcgr2b*-deficient mice can reduce anti-dsDNA, inflammatory cytokines, and glomerulonephritis^34^. However, ISD017-treated preclinical lupus-prone mice might not represent the actual clinical situation of SLE patients with overt active lupus phenotypes. Thus, we treated symptomatic lupus mice that presented with autoantibodies and proteinuria with ISD017 or the standard treatment (cyclophosphamide) and compared the efficacy and targeted effector cells of these 2 reagents.

Treatment with ISD017 and CYC reduced the mortality of *Fcgr2b*-deficient lupus mice with a comparable survival rate, suggesting that ISD017 could be a therapeutic drug for SLE. Although the mice did not show a difference in serum creatinine among the groups, only CYC reduced the urine protein creatinine ratio during the treatment period of 2 months. CYC is an alkylating agent that is cycle-cell nonspecific. It exerts a marked action against cells in the dividing phase and may also alkylate quiescent cells, which could reduce proteinuria in glomerular diseases^49^. Based on the treatment in human SLE, the duration of induction therapy for lupus nephritis is 6 months. Although the effect of ISD017 treatment did not change proteinuria (urine albumin/urine creatinine ratio), the reason could be derived from the short treatment interval in this study (2 months). If ISD017 treatment is extended longer than 2 months, the proteinuria may reduce. One limitation of our study is the omission of the total proteinuria score, recognized as a valuable indicator of lupus nephritis activity.

Some studies show that proteinuria did not correlate with renal activity because persistent protein could occur from active nephritis or inactive fibrosis. The follow-up studies of kidney biopsy in SLE patients after treatment of lupus nephritis showed that proteinuria did not predict the activity of glomerulonephritis^50–52^. Thus, we assessed the efficacy of ISD017 by measuring the histopathology of the kidney representing lupus nephritis. ISD017 and CYC significantly reduced glomerular and interstitial scores, confirming the ability of ISD017 to decrease the severity of lupus nephritis, similar to standard CYC treatment. In addition, ISD017 showed a significant reduction of IgG and C3c deposition in the glomeruli. We believe that the effect of ISD017 treatment is significant in changing renal pathology and increasing the survival of treated mice compared to untreated mice. These data suggested that the efficacy of ISD017 is at least comparable to that of CYC.

Autoantibodies in systemic lupus erythematosus (SLE) indicate impaired B cell function. This impairment can stem from intrinsic dysfunctions within B cells or indirect stimulation caused by SLE. The presence of anti-nuclear antibody (ANA) serves as an essential marker for autoimmune diseases. Detection of ANA is a screening for autoantibodies against self-antigens, which contains polyclonal specificity of autoantibodies^53^. ANA can occur after polyclonal B cell activation^54^. The ANA patterns of serum from the *Fcgr2b*^*−/−*^ mice showed homogenous and fine-speckled patterns, which did not change by the treatment. The observed homogenous patterns in the ANA staining are indicative of anti-dsDNA specificity. The presence of multiple ANA staining patterns in the *Fcgr2b*^*−/−*^ mice suggests the existence of various autoantibodies.

We opted to focus on anti-dsDNA antibodies due to their noted association with the lupus nephritis phenotype and anti-dsDNA change in a previous paper on the double deficiency of *Fcgr2b* and *Sting* mice^11^. Anti-dsDNA is a pathogenic autoantibody in lupus nephritis^55^. However, anti-dsDNA partly correlates with SLE disease activity^56^. The sensitivity of the anti-dsDNA level might not represent the lupus nephritis activity in our experiment. This result may be explained by the short duration of treatment (2 months), and the treatment did not reduce the number of plasma cells. The reduction in ANA but not anti-dsDNA and total IgG after ISD017 and CYC treatment suggested that these drugs may target specific activated B cells but not plasma cells. The observation that treatment reduced ANA levels without a corresponding decrease in anti-dsDNA suggests that the therapeutic intervention may preferentially impact autoantibodies other than anti-dsDNA. These potentially affected autoantibodies were not identified within the scope of this study, representing a limitation of our research.

The germinal center serves as the site where B cells encounter antigens presented by APCs, leading to their differentiation. Germinal center B cells actively proliferate, causing somatic hypermutation^57^. Fully differentiated plasma cells are dormant and show less proliferation^58^. Both CYC and ISD017 did not reduce the germinal center B cells (B220^+^GL7^+^FAS^+^) and plasma cells (B220^+^CD138^+^). However, only CYC reduced activated B cells (B220^+^GL7^+^). While the MFI of IAb on B cells represents the activation of B cells, we did not detect the difference in MFI between the non-treated and treated *Fcgr2b*^*−/−*^ groups. However, the number and percentage of B220^+^IAb^+^ cells were decreased by CYC treatment. The discrepancy in the IAb data could be that some B220^+^ cells showed a mixed response to the treatment, creating the IAb^hi^ and IAb^low^ cells, which may cause high variability and make MFI at an insignificant level. However, the number of IAb^+^B220^+^ cells represented B cells with IAb^hi^, which may suggest the readiness of B cells for presenting antigens to activate CD4^+^ T cells. CYC is an alkylating agent that preferentially attacks proliferating cells and has less influence on quiescent cells. The data suggested different targeted mechanisms between ISD017 and CYC. Additionally, previous research has provided evidence highlighting the crucial role of CYC, a specific substance, in regulating the expression and function of critical cells involved in B cell immunity^59^.

Effector memory T cells are the major producers of inflammatory cytokines and provide help for B cell proliferation. Moreover, T cell activation also contributes to B cell activation, leading to autoantibody secretion and causing damage to various tissues^60^. While ISD017 and CYC effectively reduced the expansion of effector memory T cells, the number of activated naïve T cells increased during CYC treatment. ISD017 decreased the expansion of CD69^+^CD4^+^ T cells but did not decrease the MFI of CD69 on CD4^+^ T cells. This data suggested that ISD017 may inhibit the differentiation of mature T cells but did not directly act on T cell activation. Given that we did not evaluate T-follicular or T-regulatory cells in our study, whether ISD017 impacts these cell types remains unclear.

CD69 is an early-activated marker, and its expression on T cells leads to cytokine production and cell proliferation^61^. The interaction between these cells and dendritic cells, facilitated by the MHC molecule, triggers the activation of CD69^+^ T cells. As a result, significant changes occur in cell function, leading to the differentiation of Th1 or Th17 cells and a notable increase in the production of various cytokines^62^. While ISD017 did not decrease anti-dsDNA levels, an earlier study reported a strong correlation between the expression levels of CD69 and the SLE disease activity index (SLEDAI) score^63^. Therefore, CD69^+^ T cells may be pathogenic, causing active disease activity. The fact that we did not test the T-follicular cells or T-regulatory cells, we could not know whether ISD017 affects these cells or not.

Granulocytes can disrupt the normal functioning of B-cell or T-cell immune cells, leading to the production of type-I interferons, tumor necrosis factor-alpha (TNFα), B-cell activating factor (BAFF), and a proliferation-inducing ligand (APRIL). The complex interaction among these factors is crucial for driving the proliferation pathways involved in SLE and regulating epigenetic expression, which can negatively affect SLE development and progression^64^. These granulocytes form neutrophil extracellular traps (NETs) when activated by autoantibodies, releasing large DNA complexes—this immune complex triggers the activation of plasmacytoid dendritic cells, the primary source of type-I interferons. Imbalances in NET production and elimination may be pivotal in advancing disease^65^.

Notably, experimental studies utilizing lupus models have provided compelling evidence supporting the link between increased expression of IL-1β and the severity and accelerated progression of the disease^66,67^. IL-1β knockout in lupus-prone NZM2328 mice still leads to the development of lupus phenotypes^68^. This finding suggested that the other signaling pathway is required for lupus development in NZM2328 mice. STING-mediated lysosomal damage activates NLRP3 inflammasome-dependent pyroptosis^69^. The reduction in IL-1β in the ISD017-treated mice should be an effect of STING inhibition in this model. Activation of the STING pathway induces type I IFN and proinflammatory cytokines, including IL-6 and TNF-α^70^. Here, we detected a reduction in interferon-inducible genes but not IL-6. IL-6 in *Fcgr2b*-deficient mice may be produced via a STING-independent pathway.

ISD-017 effectively reduced the expansion of granulocytes (Ly6c^+^Ly6g^+^) and the production of IL-1β, an inflammasome-mediated cytokine, but CYC did not. However, the kidney pathology of *Fcgr2b*-deficient mice showed NETs^40^. Netosis of neutrophils can activate complement cascade^71^. The immunofluorescence staining of C3c was significantly reduced with ISD017 but not CYC. The effect of ISD017 in rescued lupus nephritis may be derived from the reduction in neutrophils, NETs formation, and complement activation. STING can activate IRF3 and IRF7 to induce type I IFN production^72^. The absence of *Sting* in *Fcgr2b*-deficient mice decreased the expression of both *Irf3* and *Irf7* in the kidney^11^. Treatment with ISD017 and CYC targeted different interferon-inducible genes in the kidney of *Fcgr2b*^*−/−*^ mice. Both ISD017 and CYC reduced *Isg15* expression, with CYC showing more effect than ISD017. CYC suppressed *Irf3* expression better than ISD017. However, ISD017 suppressed *Irf7* and *Mx1* in the kidneys of *Fcgr2b*-deficient mice. The data suggested that ISD017 and CYC preferentially inhibited interferon-inducible genes in the kidney, which lessened glomerulonephritis in *Fcgr2b*^*−/−*^ mice.

It should be noted that this study does not provide information on long-term survival and potential long-term side effects. Further research is necessary to explore these aspects comprehensively. It would be interesting to see the effect of ISD017 in other lupus mouse models, which is beyond the scope of this study. Due to some controversies regarding STING function in mouse models and the heterogeneity of lupus disease, further study of STING function in other mouse models or human SLE is needed to clarify the beneficial role of targeting STING-mediated signaling in a specific subgroup of SLE.

In summary, ISD017 rescued lupus nephritis in symptomatic129.B6. *Fcgr2b*-deficient mice. While CYC targeted activated B cells, ISD017 targeted activated T cells and neutrophils. Since ISD017 impacts different parameters compared to cyclophosphamide, it presents a unique opportunity to be used in conjunction with cyclophosphamide in treatment protocols. This dual approach could reduce the required dosage of cyclophosphamide, thereby minimizing its side effects while maintaining therapeutic efficacy.

### Supplementary Information

Supplementary Figure 1.

## Data Availability

All relevant data have been presented in the manuscript. Requests for or questions about the data can be addressed to Prapaporn.pis@mahidol.ac.th.
