# Supplementary material for: The STING inhibitor (ISD-017) reduces glomerulonephritis in 129.B6.Fcgr2b-deficient mice
Source: Sci Rep. 2024 May 14;14:11020. doi: 10.1038/s41598-024-61597-z (PMC11094069; doi:10.1038/s41598-024-61597-z)
Supplement: Supplementary file 1 — Supplementary Figure 1. [file 41598_2024_61597_MOESM1_ESM.pdf]

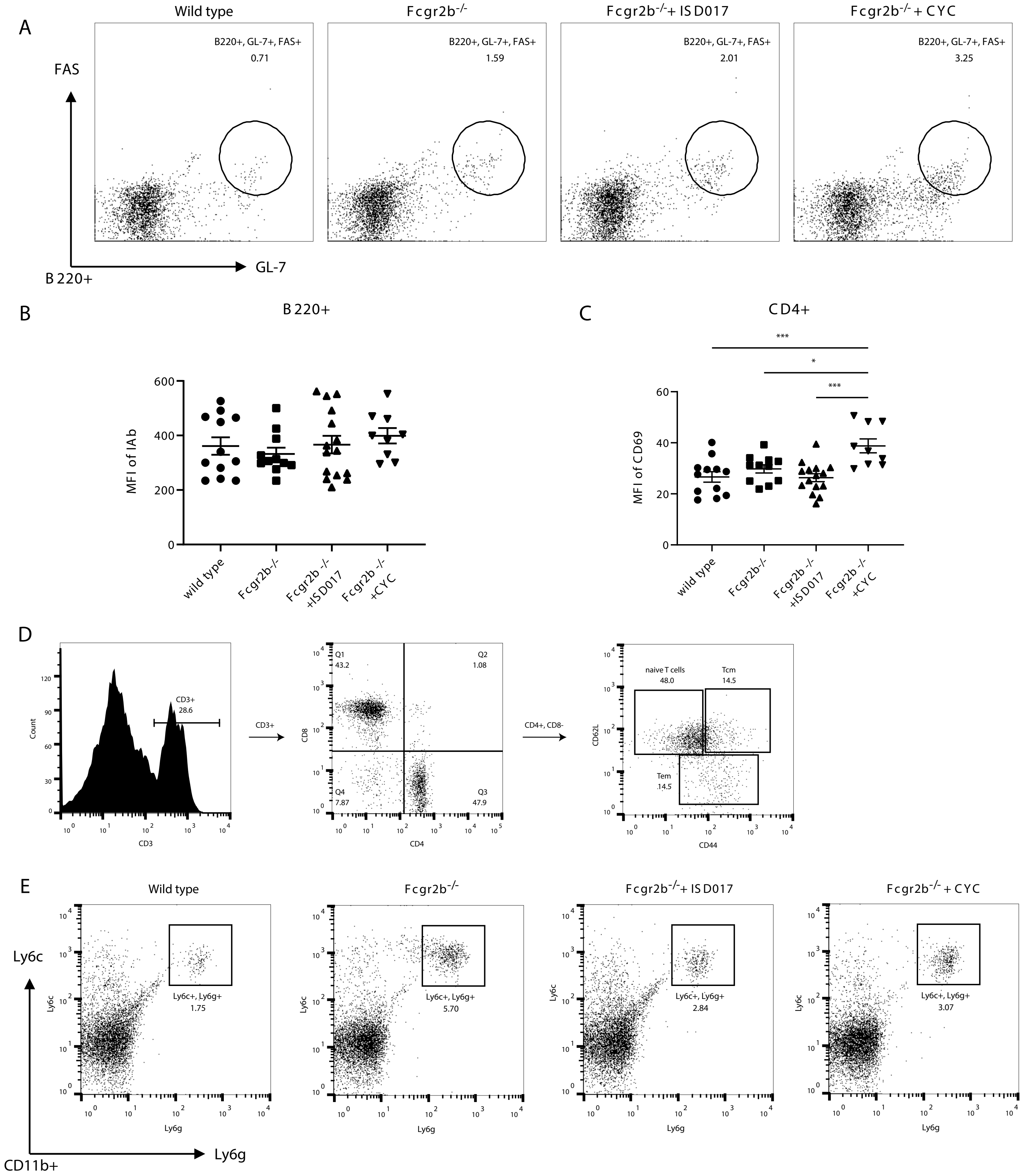

Supplementary Figure 1.

Flow cytometry analysis of isolated splenocytes from WT and *Fcgr2b*$^{-/-}$ mice.
Isolated splenocytes from *Fcgr2b*$^{-/-}$ mice (8 months old) from ISD017- or CYC-treated and control *Fcgr2b*$^{-/-}$ mice were analyzed by flow cytometry. (A) The dot plots showed the gating strategy of B220^{+}GL-7^{hi}FAS^{hi} (germinal center B cells) from the representative mice. (B) The mean fluorescence intensity (MFI) of IAb on B220^{+} cells and (C) MFI of CD69 on CD4^{+} T cells are shown. (D) The dot plots showed the gating strategy of CD44 and CD62L on CD3^{+}CD4^{+} T cells. (E) The dot plots showed the gating strategy of Ly6g and Ly6c (neutrophils) from the representative mice.
